# Intracorporeal urinary diversion offers the advantage of delaying postoperative renal function injury in patients undergoing robot-assisted radical cystectomy

**DOI:** 10.3389/fonc.2024.1435050

**Published:** 2024-09-04

**Authors:** Hao Wang, Wendi Wang, Xun Wang, Changhua Fang, Kangkang Zhao, Tianyi Chen, Chengwei Zhang, Shiwei Zhang, Hongqian Guo, Gutian Zhang

**Affiliations:** ^1^ Department of Urology, Nanjing Drum Tower Hospital, Affiliated Hospital of Medical School, Nanjing University, Nanjing, China; ^2^ Department of Urology, Medical School of Southeast University Nanjing Drum Tower Hospital, Nanjing, China

**Keywords:** bladder cancer, robot-assisted radical cystectomy, urinary diversion, acute kidney injury, chronic kidney injury

## Abstract

**Objective:**

To analyze changes in renal function and associated risk factors in patients with bladder cancer undergoing robot-assisted radical cystectomy (RARC) with intracorporeal or extracorporeal urinary diversion (ICUD or ECUD).

**Methods:**

Clinical-pathological data was extracted from electronic medical records of 266 patients with bladder cancer who underwent RARC at our institution between August 2015 and August 2022. Postoperative renal function was assessed using the estimated glomerular filtration rate (eGFR).

**Result:**

Patients were classified into ECUD and ICUD groups based on the surgical approach. Significant differences in eGFR were observed between the two groups at 1, 2, and 3 years postoperatively. Moreover, 112 patients (42.1%) experienced long-term renal function injury. Independent risk factors for long-term renal function injury included the type of surgical approach, ureteroenteric anastomotic strictures, and pathological stage T3 or above. In terms of short-term renal function, 30 cases of acute kidney injury (AKI) were observed, with an incidence rate of 11.3%. No difference in AKI incidence was found between the groups.

**Conclusions:**

Postoperative AKI and chronic kidney injury are prevalent complications following RC. This study highlights that pathological stage, ureteroenteric anastomotic strictures, and ECUD significantly impact long-term renal function, but the type of urinary diversion (ileal conduit or orthotopic neobladder) had no effect on renal function, and ICUD was superior in terms of long-term renal injury rate. Therefore, precise preoperative assessment and the selection of appropriate surgical approach are crucial for preserving renal function in patients with bladder cancer.

## 1 Introduction

Bladder cancer (BCa) is a prevalent neoplasm of the urinary system. In China, it is the fourth most common malignancies in males and tenth in females. BCa is classified into non-muscle-invasive bladder cancer and muscle-invasive bladder cancer. For locally muscle invasive bladder cancer without distant metastasis, the standard treatment involves cisplatin-based neoadjuvant chemotherapy followed by radical cystectomy (RC), which also applies to certain high-risk and very high-risk non-muscle-invasive cases (1, 2). With the advent of robotic-assisted laparoscopic surgical systems in China, robot-assisted radical cystectomy (RARC) has seen increased adoption and has become the preferred method in numerous medical centers (3). Initially, most surgeons performed extracorporeal urinary diversion (ECUD). However, the use of intracorporeal urinary diversion (ICUD) is steadily increasing. Comparative studies, both domestic and international, have reported no significant differences between RARC with ICUD and RARC with ECUD in terms of surgical duration, positive margin rate, number of lymph nodes retrieved, and survival rates. Additionally, RARC with ICUD is associated with less blood loss and faster bowel recovery (4, 5). Postoperative renal injury incidence varies from 20% to 70% across different surgical approaches (6–8). This variation is likely due to differences in definitions, measurements, and follow-up periods across studies. However, regardless of the surgical approaches, most patients experience renal injury, which is linked to increased hospitalization rates, all-cause mortality, and cancer-specific mortality (9). While several studies have evaluated long-term renal function changes following open radical cystectomy (ORC), a single institution in the United States has compared long-term renal function outcomes and progression to CKD stages 3B and higher after intracorporeal RARC, extracorporeal RARC, and ORC (10). However, the risk factors for renal injury following RARC remain contentious, and there is a paucity of research comparing ICUD with ECUD in China.

To address these gaps, this study conducts a retrospective analysis of patient data undergoing RARC at the Affiliated Drum Tower Hospital of Nanjing University Medical School. We aim to compare perioperative outcomes, renal function changes, and associated risk factors between different urinary diversion methods and surgical approaches.

## 2 Methods

We reviewed the data of 266 patients who underwent RARC at the Department of Urology, Affiliated Drum Tower Hospital of Nanjing University Medical School, from August 2015 to August 2022. We collected demographic data and perioperative outcomes including surgical duration, estimated blood loss, transfusion rate, pathological stage, and clinical parameters potentially affecting postoperative renal function. This study evaluates the surgical approaches, renal function changes, and factors influencing renal function changes.

Inclusion criteria: (1) Patients with a preoperative diagnosis of locally resectable muscle-invasive bladder cancer (T2-4a, N0-x, M0) or high-risk non-muscle-invasive bladder cancer. (2) Patients who underwent RARC.

Exclusion criteria: (1) Patients undergoing bladder cystectomy for reasons other than bladder cancer, with or without nephroureterectomy. (2) Patients with a preoperative estimated glomerular filtration rate (eGFR) ≤45 mL/min/1.73m^2^ or severe hydronephrosis. (3) Patients with incomplete demographic data or those who did not participate in postoperative follow-up.

eGFR was calculated using the CKD-Epidemiology Collaboration formula (11). Baseline eGFR was determined from a single preoperative measurement, while postoperative eGFR was recorded by surgeons during follow-up, with a minimum follow-up period of at least one year. Ureteroenteric anastomotic strictures were identified as asymptomatic or symptomatic hydronephrosis in patients with radiological evidence of obstruction at the ureterointestinal anastomosis, primarily based on CT urography or confirmed during surgery. Long-term renal injury was defined as a reduction in eGFR of ≥10 mL/min/1.73m^2^ from baseline (10, 12). Acute kidney injury (AKI) was defined according to KDIGO guidelines as an increase in serum creatinine to more than 1.5 times the baseline value, with this increase occurring or presumed to have occurred within the preceding seven days (13). All patients receiving neoadjuvant and adjuvant chemotherapy were treated with a gemcitabine and cisplatin regimen, with neoadjuvant chemotherapy administered every three weeks for 3-4 cycles and adjuvant chemotherapy every three weeks for 4-6 cycles. Postoperative follow-up was conducted every three months in the first year and at least annually thereafter.

Continuous variables were presented as mean ± standard deviation and compared between groups using an independent sample t-test. Categorical variables were presented as frequencies (percentages) and compared using the chi-square test. Logistic regression analysis was used to identify factors influencing renal function. All statistical analyses were performed using SPSS version 25.0, with two-tailed p-values < 0.05 considered statistically significant.

## 3 Result

### 3.1 Baseline characteristics

Patients were categorized into two groups based on their surgical approach for urinary diversion: ECUD and ICUD groups. The study included 266 patients with a follow-up duration of 36 months. Among these, 84 underwent orthotopic neobladder (ON) reconstruction and 182 underwent ileal conduit (IC) reconstruction. The cohort comprised 232 males and 34 females. Demographic and clinical characteristics are detailed in **Table 1**. In the ECUD and ICUD groups, the majority of patients underwent IC (68.4% *vs*. 31.6%, *P*= 0.086). The baseline estimated glomerular filtration rate (eGFR) for all patients was 107.3 ± 23.3 mL/min/1.73m^2^ (ECUD *vs*. ICUD: 104.7 ± 24.1 *vs*. 107.5 ± 23.0, *P*=0.377), with preoperative creatinine levels of 70.1 ± 16.5umol/L and 69.5 ± 15.9umol/L respectively (ECUD *vs*. ICUD, *P*=0.770). Neoadjuvant chemotherapy was administered to 19.5% of patients, with no statistically significant difference in the distribution of CKD stages 1-3a between the two groups preoperatively (CKD stage 1: 79.7%, CKD stage 2: 17.7%, CKD stage 3a: 2.6%, *P*=0.637). Among the cases, there were 84 ON and 182 IC reconstructions, with 65 (34.8%) and 122 (65.2%) ICUD and 19 (24.1%) and 60 (75.9%) ECUD, respectively. Notably, significant differences in bladder reconstruction methods between the two groups was not observed (*P*=0.086).

**Table 1:** Preoperative patient characteristics.

	ECUD	ICUD	Total	*P* value
Number of patients (%)	79(29.7)	187(70.3)	266(100)	--
Age (years)	69.0 ± 9.6	66.7 ± 9.3	67.4 ± 9.4	0.079
BMI (kg/m^2^)	25.6 ± 2.0	25.8 ± 1.8	25.7 ± 1.8	0.596
Females (%)	14(17.7)	20(10.7)	34(12.8)	0.117
Hypertension (%)	28(35.4)	58(31.0)	86(32.3)	0.481
Diabetes (%)	19(4.1)	46(24.6)	65(24.4)	0.924
Smoking history (%)	11(13.9)	29(15.5)	40(15.0)	0.741
Drinking history (%)	14(17.7)	38(20.3)	52(19.5)	0.625
Neoadjuvant chemotherapy (%)	16(20.3)	36(19.3)	52(19.5)	0.851
Adjuvant chemotherapy (%)	6(7.6)	13(7.0)	19(7.1)	0.852
IC (%)	60(75.9)	122(65.2)	182(68.4)	0.086
ON (%)	19(24.1)	65(34.8)	84(31.6)	0.086
Preoperative CKD stage 1 (%)	61(77.2)	151(80.7)	212(79.7)	0.637
Preoperative CKD stage 2 (%)	15(19.0)	32(17.1)	47(17.7)	0.637
Preoperative CKD stage 3a (%)	3(3.8)	4(2.1)	7(2.6)	0.637

### 3.2 Perioperative outcomes and complications

ICUD was associated with a longer operative time compared to ECUD (*P*=0.080), although this difference was not statistically significant. Estimated blood loss was significantly lower in the ICUD group compared to the EUCD group (303.5 ± 116.5 *vs*. 379.8 ± 233.3 mL, *P*=0.007). However, transfusion rates did not differ significantly between the groups (ECUD *vs*. ICUD 24.1% *vs*. 20.3%, *P*=0.498). There were no significant differences in the distribution of pathological T3 stage or higher (*P*=0.987) and lymph node positivity rate (*P*=0.826) between the two groups. The incidence of ureteroenteric anastomotic strictures was lower in the ICUD group compared to the ECUD group, though this difference was not statistically significant (ICUD: 8.6%; ECUD: 16.5%, *P*=0.059), as shown in **Table 2**.

**Table 2:** Perioperative outcomes and complications.

Variable	ECUD (n=79)	ICUD (n=187)	Total (n=266)	*P* value
IC(%)	60(75.9)	122(65.2)	182(68.4)	0.086
ON(%)	19(24.1)	65(34.8)	84(31.6)	0.086
Operative time in minutes (min)	400.4 ± 68.2	417.8 ± 84.1	412.6 ± 80.0	0.080
Estimated blood loss (mL)	379.8 ± 233.2	303.5 ± 116.5	326.1 ± 163.6	0.007
Pathologic T3 disease ≥ (%)	24(30.4)	57(30.5)	81(30.5)	0.987
Node positive disease (%)	17(21.5)	38(20.3)	55(20.7)	0.826
Postoperative CKD stage 3B> (%)	9(11.4)	3(1.1)	12(4.5)	<0.001
Experienced eGFR drop ≥10 mL /min/1.73m^2^ (%)	50(63.3)	62(33.2)	112(42.1)	<0.001
Ureteroenteric anastomotic strictures (%)	13(16.5)	16(8.6)	29(10.9)	0.059

Overall, 37.6% of patients experienced complications within the first 30 days postoperatively, with 35.3% classified as minor complications (Grade I-II) and 2.3% as major complications (Grade III-V). The most common early complication was urinary tract infection (UTI), affecting 34 patients (12.8%), with 38.2% of these receiving intravenous antibiotic treatment. The incidence of late complications (beyond 90 days) was 38.7%, with hydronephrosis (21.4%) and UTI (17.7%) being the most frequent. None of the patients with late complications experienced major complications.

### 3.3 Renal function outcomes

**Figure 1:**
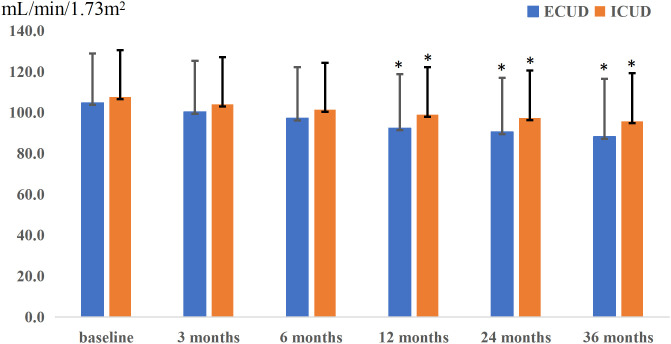
Postoperative renal function changes in two groups, presenting the trend of renal function changes over time for patients undergoing different surgical approaches. The horizontal axis represents follow-up time, while the vertical axis represents the estimated glomerular filtration rate (eGFR). The extracorporeal urinary diversion (ECUD) group is depicted in blue, and the intracorporeal urinary diversion (ICUD) group is depicted in orange. Additionally, error bars are represented by black lines. The * indicates that the P-value is less than 0.05.

Renal function was monitored at specified intervals through eGFR measurements (**Figure 1**). There was no significant difference in the average preoperative eGFR between the two groups. Postoperative eGFR values for the ECUD group were 100.3 ± 25.0, 97.1 ± 25.3, 92.3 ± 26.5, 90.4 ± 26.5, and 88.1 ± 28.3 mL/min/1.73m^2^ at 3, 6, 12, 24, and 36 months, respectively. For the ICUD group, the values were 103.9 ± 23.1, 101.3 ± 23.0, 98.9 ± 23.3, 97.3 ± 23.3, and 95.7 ± 23.5 mL/min/1.73m^2^ at the same points. Significant differences in eGFR between the two groups were observed at 1 (*P*=0.042), 2 (*P*=0.034), and 3 years (*P*=0.037) postoperatively. During the follow-up period, 112 patients (42.1%) experienced long-term renal injury, with 12 cases (4.5%) progressing to CKD3B or higher. Of these, nine cases (11.4%) were in the ECUD group and three cases (1.1%) were in the ICUD group, with a statistically significant difference (*P*<0.001). Patients were classified into renal injury and non-renal injury groups based on the presence of long-term renal injury. Univariate analysis revealed significant differences between the two groups in terms of age (*P*=0.007), surgical approach (*P*<0.001), ureteroenteric anastomotic strictures (*P*<0.001), pathological stage T3 or higher (*P*<0.001), postoperative hydronephrosis (*P*=0.006), and blood loss (*P*=0.007). Multivariate logistic regression analysis revealed that different surgical approaches (OR 0.24, 95% CI: 0.12-0.46, *P*< 0.001), ureteroenteric anastomotic strictures (OR 4.37, 95% CI: 1.32-14.45, *P*= 0.016), and pathological stage T3 or higher (OR 6.21, 95% CI: 3.20-12.07, *P*<0.001) were independent risk factors for long-term renal injury postoperatively (**Tables 3**, **4**).

**Table 3:** Predictors of long-term renal impairment after RARC.

Variables	Total (n=266)	With renal Injury (n=112)	Without renal Injury (n=154)	Univariate *P* value	Multivariate *P* value
Age(years)	67.4 ± 9.4	69.2 ± 9.7	66.1 ± 9.0	0.007	0.108
Female (%)	34(12.8)	19(17.0)	15(9.7)	0.081	0.397
BMI	25.7±	25.7±	25.7±	0.891	--
(kg/m^2^)	1.8	1.9	1.8		
UD				0.368	0.362
ON	84(31.6)	32(28.6)	52(33.8)		
IC	182(68.4)	80(71.4)	102(66.2)		
Surgical approaches				<0.001	<0.001
ICUD	187(70.3)	62(55.4)	125(81.2)		
ECUD	79(29.7)	50(44.6)	29(18.8)		
UES	29(10.9)	24(21.4)	5(3.2)	<0.001	<0.001
Preoperative CKD stage				0.387	--
Stage1	212(79.7)	85(75.9)	127(82.5)		
Stage2	47(17.7)	24(21.4)	23(14.9)		
Stage3a	7(2.6)	3(2.7)	4(2.6)		
Pathologic T3 disease ≥ (%)	81 (30.5)	58 (51.8)	23 (14.9)	<0.001	<0.001
Hypertension (%)	86 (32.3)	36 (32.1)	50 (32.5)	0.955	--
Diabetes (%)	65 (24.4)	28 (25.0)	37 (24.0)	0.855	--
Neoadjuvant chemotherapy (%)	52 (19.5)	21 (18.8)	31 (20.1)	0.779	--
Adjuvant chemotherapy (%)	19 (7.1)	9 (8.0)	10 (6.5)	0.630	--
urinary tract infection (%)	47 (17.7)	20 (17.9)	27 (17.5)	0.945	--
Hydronephrosis (%)	57 (21.4)	30 (26.8)	27 (17.5)	0.069	0.593
Transfusion (%)	63 (23.7)	36 (32.1)	27 (17.5)	0.006	0.058
Operative time (min)	412.6±80.0	410.7±83.2	414.1±77.9	0.733	--
Estimated blood loss (mL)	326.1±163.6	360.3±207.0	301.3±123.5	0.007	0.384

**Table 4:** univariate and multivarite logistic regressions analysis to predict long-term renal impairment after RARC.

Variables	Univariate			Multivariate		
	OR	95%CI	*P* value	OR	95%CI	*P* value
Age(years)	1.026	1.002-1.062	0.007	1.652	0.904-3.021	0.108
Female (%)	0.698	0.295-1.651	0.081	0.671	0.273-1.650	0.397
BMI	1.041	0.879-1.232	0.891			
UD	1.057	0.540-2.068	0.368	1.354	0.689-2.659	0.362
Surgical approaches	0.393	0.208-0.744	<0.001	0.236	0.122-0.458	<0.001
UES	24.444	9.547-62.589	<0.001	4.485	1.336-15.056	<0.001
Preoperative CKD stage	1.322	0.724-2.413	0.387			
Pathologic T3 disease ≥ (%)	5.752	2.970-11.143	<0.001	6.172	3.172-12.012	<0.001
Hypertension (%)	0.907	0.464-1.774	0.955			
Diabetes (%)	1.302	0.650-2.608	0.855			
Neoadjuvant chemotherapy (%)	0.910	0.411-2.019	0.779			
Adjuvant chemotherapy (%)	1.648	0.564-4.813	0.630			
urinary tract infection (%)	0.891	0.387-2.049	0.945			
Hydronephrosis (%)	1.754	0.889-3.458	0.069	1.963	0.959-4.020	0.593
Transfusion (%)	2.081	1.047-4.137	0.006	1.253	0.548-2.866	0.058
Operative time (min)	0.999	0.996-1.003	0.733			
Estimated blood loss (mL)	1.002	1.001-1.004	0.007	1.001	0.999-1.004	0.384

OR, Odds Ratio; CI, Confidence Interval; BMI, Body Mass Index; UD, Urinary Diversion; UES, ureteroenteric anastomotic stricture; CKD, Chronic Kidney Disease.

Regarding short-term postoperative renal function, 30 patients (11.3%) experienced AKI during the follow-up period, with 20% in the ECUD group and 80% in the ICUD group (*P*= 0.217). No statistically significant differences in parameter were observed among patients with AKI, as shown in **Table 5**.

**Table 5:** Perioperative and postoperative indicators based on the occurrence of AKI.

Variable	With AKI(n=30)	Without AKI(236)	Total(n=266)	*P* value
Age(years)	70.1 ± 8.9	67.0 ± 9.5	67.4 ± 9.5	0.099
Females (%)	2 (6.7)	32 (13.6)	34 (12.8)	0.287
BMI (kg/m^2^)	25.3 ± 1.9	25.8 ± 1.8	25.7 ± 1.8	0.215
ICUD (%)	24 (80.0)	163 (69.1)	187 (70.3)	0.217
ECUD (%) ON (%) IC (%)	6 (20.0) 15 (50.0) 15 (50.0)	73 (30.9) 69 (29.2) 167 (70.8)	79 (29.7) 84 (31.6) 182 (68.4)	0.217 0.021 0.021
Operative time (min)	436.3±72.0	409.6± 80.6	412.6±80.0	0.085
Estimated blood Loss(mL)	325.0± 145.5	326.3± 166.0	326.1±163.6	0.968
Pathologic T3 disease ≥ (%)	10 (33.3)	71 (30.1)	81 (30.5)	0.716
Node positive disease (%)	5 (16.7)	50 (21.2)	55 (20.7)	0.565
Preoperative creatinine value (umol/L)	71.2 ± 17.2	69.5 ± 15.9	69.7 ± 16.0	0.589
Preoperative eGFR(mL/min/1.73m^2^)	107.4 ± 31.9	107.4 ± 23.6	106.6 ± 23.3	0.995
Transfusion (%)	8 (26.7)	49 (20.8)	57 (21.4)	0.458

## 4 Discussion

In this study, 4.5% of patients progressed to CKD3B or higher stages of CKD following RARC, a proportion lower than some reported data (7). This difference may be attributed to the higher baseline renal function of patients in our study compared to those in international studies. Additionally, 42.1% of patients experienced postoperative renal function injury, which aligns with or slightly lower than findings from other retrospective studies. Variations in these outcomes may be attributed to differences in inclusion/exclusion criteria, such as excluding patients with severe preoperative hydronephrosis, variations in follow-up duration, or differences in renal function assessment methods. Nonetheless, these findings underscore that a considerable proportion of patients experience renal function injury following RARC.

Our study confirms that ECUD is an independent risk factor for long-term renal function injury following RARC. Although there were no significant differences in preoperative eGFR or at 3 and 6 months postoperatively between the two surgical approaches, significant differences emerged at 1, 2, and 3 years postoperatively, with the ECUD group showing a more rapid decline in eGFR compared to the ICUD group. Razdan et al. previously reported that ICUD has a protective effect against eGFR decline at 24 months postoperatively (14). Our study extends these findings by demonstrating that ECUD remains a significant risk factor for long-term renal function injury over a longer follow-up period. Given that RARC with ICUD is associated with less blood loss and no significant differences in operative time, positive margin rate, lymph node yield, and survival rates compared to RARC with ECUD, it is recommended that medical facilities with the capability should consider prioritizing RARC with ICUD as the preferred surgical approach.

Eisenberg et al. identified postoperative ureteroenteric anastomotic strictures as an independent risk factor for renal function impairment following ORC in a cohort of 1631 patients treated at a single institution from 1980 to 2006 (12). Similarly, our study corroborates this finding. The lack of corresponding force feedback in robotic surgical systems may lead to excessive ureteral mobilization during anastomoses in robot-assisted surgery. In ECUD, excessive stretching of the ureters compromises their blood supply. Desai et al. conducted a prospective study involving 132 patients undergoing RARC with intracorporeal IC urinary diversion, reporting a stricture rate of 3.8% at 90 days postoperatively (15). This stricture rate is lower than the 9.4% reported by Anderson et al, suggesting that ICUD, which requires a shorter length of the ureter, may reduce the likelihood of excessive dissection and subsequent ureteral adventitia. Excessive dissection can lead to compromised ureteral blood supply, leading to ischemia at the anastomotic site, perianastomotic fibrosis, and scar formation, resulting in ureteroenteric anastomotic strictures. These structures can further cause ureteral and renal pelvis dilation and hydroureteronephrosis, leading to long-term renal function injury (16). Currently, there is limited evidence on how factors such as urinary diversion, choice of intestinal segment, surgical approaches, ureteroenteric anastomosis, and suturing techniques influence stricture outcomes.

In addition to identifying ureteroenteric anastomotic strictures as an independent risk factor for long-term renal function injury postoperatively, this study also found that pathological stages at T3 or higher are an independent risk factor for long-term renal injury. This association may be due, in part, to the postoperative adjuvant therapy received by patients at this stage, as 19.5% of those with pathological stages at T3 or higher received such treatment. Adjuvant therapy, typically including gemcitabine plus cisplatin (GC) chemotherapy, may impact renal function. Further analysis revealed that the incidence of postoperative hydronephrosis was 35.8% in the T3 or higher group compared to 18.4% in the T2 or lower group, with a statistically significant difference observed. Therefore, the influence of pathological stage at T3 or higher on long-term renal function injury may be multifactorial. Additionally, Nishikawa et al. identified infection-related complications, such as pyelonephritis, as risk factors for renal function impairment in a retrospective analysis of 169 patients (6). In our study, 17.7% of patients experienced postoperative UTI; however, specific data on pyelonephritis were not recorded, largely due to follow-up at other medical institutions. Identifying and managing complications following RC is crucial. Our findings indicate that 37.6% of patients experienced complications within 30 days postoperatively, of which 2.3% were high-grade. The incidence of late complications was 38.7%, with no major complications reported among patients with late complications. These results are consistent with or slightly lower than those reported in previous studies (17, 18).

Regarding short-term postoperative renal function, 11.3% of patients experienced AKI, with no significant difference in AKI incidence between ICUD and ECUD. Hyllested et al. demonstrated that RARC is associated with a higher risk of AKI compared to ORC in a study of 755 patients (19). The learning curve associated with RARC and its representation of only 22% of surgeries during their study period may contribute to the higher risk of AKI. Furrer et al. also reported an increased risk of AKI in patients with surgical time exceeding 400 minutes (20). However, this study did not identify specific factors influencing AKI.

This study has several limitations. It is a retrospective analysis and did not collect data prospectively, which may affect the accuracy of the results. Additionally, non-nadir eGFR values obtained during postoperative follow-up may influence the findings. Furthermore, patients undergoing benign or palliative cystectomy, as well as those with preoperative CKD stages 3B, 4, and 5 who may experience renal function injury postoperatively, were excluded from this study, limiting the generalizability of the findings to these groups. RARC at our institution was performed primarily by three experienced urologists, each having completed a minimum of 15 RARC procedures, typically supported by a consistently reliable team. Nevertheless, variations in surgical techniques and learning curves among practitioners could still impact outcomes. Finally, due to the limited number of patients and the short postoperative follow-up period, further prospective studies with multicenter participation, larger sample sizes, and longer follow-up durations are needed.

## 5 Conclusion

Postoperative AKI and long-term renal function impairment are common complications following RC for bladder cancer, significantly affecting patients’ quality of life. Long-term follow-up is essential for the early detection and effective management of these complications. This study demonstrates that tumor characteristics (pathological stage), surgery-related complications (ureteroenteric anastomotic strictures), and the choice of surgical approaches (ECUD and ICUD) can impact long-term renal function. But the type of urinary diversion (IC or ON) had no effect on renal function, and ICUD was superior in terms of long-term renal injury rate. Therefore, precise preoperative assessment and careful selection of appropriate surgical approaches are crucial for preserving renal function in patients with bladder cancer.

## Data Availability

The original contributions presented in the study are included in the article/Supplementary Material. Further inquiries can be directed to the corresponding author.
